# Development of a Short Version of the Visual Function Questionnaire Using Item-Response Theory

**DOI:** 10.1371/journal.pone.0073084

**Published:** 2013-09-12

**Authors:** Shunichi Fukuhara, Takafumi Wakita, Masakazu Yamada, Yoshimune Hiratsuka, Joseph Green, Kotaro Oki

**Affiliations:** 1 Department of Epidemiology and Healthcare Research, Graduate School of Medicine and Faculty of Medicine, Kyoto University, Kyoto, Japan; 2 iHope International, Kyoto and Tokyo, Japan; 3 Faculty of Sociology, Kansai University, Osaka, Japan; 4 Division for Vision Research, National Institute of Sensory Organs, National Tokyo Medical Center, Tokyo, Japan; 5 Department of Ophthalmology, Juntendo University, Tokyo, Japan; 6 Graduate School of Medicine and Faculty of Medicine, University of Tokyo, Tokyo, Japan; 7 Oki Eye Surgery Center, Tokyo, Japan; Massachusetts Eye & Ear Infirmary, Harvard Medical School, United States of America

## Abstract

**Purpose:**

In clinical ophthalmology as in other fields, measuring patient-reported outcomes imposes a burden on patients. To decrease that burden, we used item-response theory (IRT) to develop and test a short version of the National Eye Institute's Visual Function Questionnaire (VFQ).

**Methods:**

We analyzed VFQ data from 276 adults in Japan. Most of them had glaucoma, cataract, or macular degeneration. Their visual acuity (Snellen fraction) averaged 20/120 (range: 20/13 to 20/2000) for the better eye, and 20/200 (range: 20/13 to 20/2000) for the worse eye. We used a polytomous IRT model, the Generalized Partial Credit Model as implemented in software for parameter scaling of rating data (PARSCALE). To select items for inclusion in the short version we examined each item's location on the latent-trait continuum, its slope, and its frequency of missing data. We also ensured representation of all 7 domains that are important in Japan. To examine the characteristics of the resulting scale, we computed its test information (an index of precision that can vary with the value of the latent trait), and carried out validation testing.

**Results:**

From 32 of the original VFQ items, we selected 11. The scale comprising those 11 items (the VFQ-J11) had test information greater than 9 for values of the latent trait between −2.0 and +0.8. The item thresholds were well-targeted for patients with vision problems. Scores on the VFQ-J11 correlated strongly and in the expected direction with measures of visual field and corrected visual acuity. As expected for a valid measure, those scores also improved by a large amount (almost one standard deviation) after cataract surgery.

**Conclusion:**

This 11-item instrument can provide reliable and the valid data on visual functioning in patients with ophthalmic problems. It is expected to be less of a burden on respondents, while it maintains good psychometric properties.

## Introduction

The Visual Function Questionnaire (VFQ) was originally developed by the National Eye Institute [1]–[3]. It is intended to measure visual functioning in 11 domains: general vision, ocular pain, near activities, distance activities, vision-specific social functioning, vision-specific mental health, vision-specific role difficulties, vision-specific dependency, driving, color vision, and peripheral vision. The 25-item version of the VFQ has been used to measure visual functioning in many contexts [4]–[7].

Marella [8] and also Pesudovs [9] used Rasch analysis with VFQ data, and they found evidence of multidimensionality. While the items were not optimally targeted to the patients, based on the fit of the data to the Rasch model, Pesudovs et al. [9] were able to recommend versions with fewer items than the original: a 6-item visual functioning scale and a 7-item socioemotional scale. For the Japanese version of the VFQ, Suzukamo, et al. [10] pointed out that the “driving” domain might not be appropriate, because the activity it asks about is relatively unimportant to some patients in Japan. They also found that the VFQ could be unidimensional only if it did not include the “color vision,” “peripheral vision,” and “ocular pain” domains. They computed scores for three Japanese versions: one with all 11 domains, one with all but the “driving” domain, and one with all but the “driving,” “color vision,” “peripheral vision,” and “ocular pain” domains. Their results indicated that the latter of those three, that is, the one with only 7 of the 11 original domains, provided the results that were most responsive to cataract surgery [10]. The Japanese-language VFQ25 has been tested [10], and has been used in a number of studies of vision-related quality of life [11]–[16].

When developing instruments such as the VFQ, there are important advantages to using item-response theory (IRT) rather than classical test theory (CTT). Unlike CTT, IRT allows characteristics of the items to be distinguished from characteristics of the respondents. Analyses based on IRT provide more detailed information about individual items, and each item can be evaluated on the same dimension as the latent trait. In addition, estimates of reliability that are based on CTT provide only a single value for the reliability of the scale as a whole, whereas analysis based on IRT provides information about how reliability varies throughout the measured range of the latent trait [17].

For patients in clinical settings, responding to many questions about visual function, sometimes via an interviewer, can impose a substantial burden. Our goals were to minimize that burden by developing a version with fewer questions, and to ensure that the decisions to delete questions were informed by evidence regarding the effects of those deletions on the instrument's psychometric properties. Therefore, using analyses based on IRT, we developed a version of the VFQ that imposes less of a burden without substantially sacrificing either precision or accuracy.

## Methods

The original version of the VFQ had 39 items in its 11 domains [3]. Fourteen of those items were considered to be optional [3], so the commonly used form of that instrument has been the VFQ25. For the present study we used data collected previously [10] from 276 adults in Japan.

Before analyzing the data we reversed the scoring where necessary so that higher scores indicated better visual functioning. Also, the item asking about reading newspapers allows participants to respond that they do not read newspapers for reasons other than visual difficulty, and we coded that response choice as missing data.

Next, to study the dimensionality of the instrument we began with data from 36 items: the VFQ-39 minus the 3 items in the “driving” domain, which were deleted because of their inappropriateness in Japan, as noted above. Using data from those 36 items, we did factor analysis (principal factor method), and examined the resulting scree plot of eigenvalues, which indicated unidimensionality. We then estimated the parameters important in IRT. For that we used a polytomous model, the Generalized Partial Credit Model [18] as implemented in software for parameter scaling of rating data (PARSCALE [19]).

### Ethical considerations

Attending physicians explained the research and ethical considerations to the participants, who then indicated their understanding by signing an informed-consent form. This work was approved by the Review Board of the Institute for Health Outcomes and Process Evaluation Research (iHope International, Kyoto and Tokyo, Japan).

### Choosing items

When choosing items to include in the short version we took into account the percentages of missing data, and the slope and location parameters estimated according to IRT. For each item, the slope parameter indicates item discrimination (precision of measurement). In general, items with high slope parameters are preferred. The location parameter indicates item “difficulty”. In the present context, “difficulty” refers to the value of the latent trait for which an item gives the most information. For example, a question asking whether one needs help from other people because of vision problems might give much information about people whose vision is severely impaired but not much information about people whose vision is only slightly impaired. In contrast, a question about reading newspapers might give much information about people whose vision is slightly impaired but not much information about people whose vision is severely impaired. Those two questions would have different “difficulties,” that is, different locations. Using the location parameter to choose items for a short-form instrument requires knowledge about the purpose of the measurement. Because we aimed to make an instrument that would be useful in clinical settings we were sure to include items of relatively high “difficulty” (i.e., locations below zero). We also chose at least one item from each of the 7 domains (general vision, near activities, distance activities, vision-specific social functioning, vision-specific mental health, vision-specific role difficulties, and vision-specific dependency).

### Reliability (precision of measurement)

To quantify reliability as it is defined in CTT, we used Cronbach's alpha. Because alpha applies only to the scale as a whole, and not to individual items, it does not easily accommodate differences in reliability at different levels of the latent trait. From the perspective of IRT, the single value of α computed for a scale can be seen as only an average, while in groups of patients the latent trait (theta) has many different values. An IRT-based index comparable to the classical measures of reliability is test information, I(theta). Specifically, the standard error of the estimated value of the latent trait is equal to the inverse of the square root of the test information. By making a graph with the latent trait (i.e., theta) on the abscissa and test information on the ordinate, one can see how the precision of the measurement varies with the value of the latent trait, which is, in this case, visual functioning. To find out how much of the precision of the original version was retained in the shorter version, we constructed such a graph for both versions. To convert between scale scores and theta, we use the following equation: theta  =  (score – mean score)/standard deviation.

All methods for estimating reliability have strengths and weaknesses. Both Cronbach's alpha and IRT-based estimates are biased by correlated errors, redundancy, and local dependence. Unlike IRT-based estimates, Cronbach's alpha is also inflated in tests with very large numbers of items, even when the reliability of each is poor. IRT is preferable to CTT, but the latter is still widely taught and used, and we computed Cronbach's alpha so that those who are more familiar with it can see how it relates to the results of IRT-based analyses.

### External criteria and comparisons with the 25-item version

For validation testing, we measured visual acuity and visual field, and we studied associations between scores on the short version and causes of visual impairment (glaucoma, cataracts, and age-related macular degeneration). For the data on visual acuity and visual field we computed correlation coefficients, and for the data on diagnosis-related differences we used one-way analysis of variance.

To study how scores on the short version change with a clinical intervention, we collected data before and after cataract surgery. These data were obtained from 14 patients who had no vision-related abnormality other than cataract. Within one month before surgery and again within one month after surgery, the patients responded to the short version of the VFQ and to the European Quality of Life-5 Dimensions scale (EQ-5D [20], [21]). The differences between the preoperative and postoperative scores on each instrument were expressed as standardized effect sizes, specifically, Cohen's d [22]. Cohen's d is the difference divided by the standard deviation of the scores at baseline. Thus, it expresses change in standard-deviation units.

## Results

### Participants

We analyzed data from 276 adults (141 women and 135 men) in Japan [10]. They ranged in age from 21 to 95 years (mean 66.8, SD 14.3). Their visual acuity (Snellen fraction) averaged 20/120 (range: 20/13 to 20/2000) for the better eye and 20/200 (range: 20/13 to 20/2000) for the worse eye. Among them were 96 who had age-related cataract, 69 who had glaucoma, 80 who had age-related macular degeneration, and 31 without a chronic ophthalmologic disease.

In 14 patients we measured the responsiveness to the effects of corrective surgery. Their mean age was 75 years (SD 9.2 years). Their corrected visual acuity ranged from 0.02 to 0.80 (Snellen's decimal unit) before surgery, and from 0.5 to 1.0 after surgery. Before surgery, the mean (± SD) of the refractions (the spherical equivalent in diopters: D) was −2.3±5.1 D, and the range was from −17 to +5 D. After surgery, the mean (± SD) was −1.2±1.3 D and the range was from −4 D to +1 D.

### Factor analysis

For factor analysis, we began with data from 36 items. These were the 25 items of the VFQ25, plus the 14 optional items, minus 3 items related to driving. Factor analysis (principal factor method) revealed seven factors with eigenvalues greater than 1: 18.35, 2.43, 2.17, 1.91, 1.57, 1.22, and 1.12. The steep drop between the eigenvalues of the first and second factors, together with the very gradual decrease from the second factor on, indicated a unidimensional structure. The factor loadings shown in Table 1 were computed on that basis of that unidimensionality. Factor loadings less than 0.4 were found for 7 items: 1 near-vision item, 2 general-health items, 2 ocular-pain items, 1 peripheral-vision item, and 1 color-vision item. Of those, the latter 6 were deleted. Item #5 (near vision, factor loading  = 0.282) was not deleted, as the other near-vision items had quite high factor loadings. The resulting 32 items formed the pool from which the short-form items were chosen.

**Table 1:** Estimates of item parameters.

Item number	Domain	Factor loading	Slope	Location	Model fit**	Missing (%)
2	General vision	.738	0.858	0.168	0.952	0.7
3	Well-being, distress	.828	0.752	0.093	0.068	0.4
5	Near vision	.863	1.066	−0.153	0.021	5.8
6	Near vision	.760	0.839	−0.125	0.204	13.0
7	Near vision	.815	1.265	−0.380	0.011	28.3
8	Distance vision	.837	0.904	−0.365	0.405	4.0
9	Distance vision	.745	0.642	−0.018	0.445	8.7
11	Social functioning	.767	1.066	−0.677	0.115	14.5
13	Social functioning	.708	0.931	−0.841	0.109	9.1
14	Distance vision	.868	1.643	−0.112	0.112	31.9
17	Role limitation	.928	1.056	−0.711	0.110	0.4
18	Role limitation	.860	0.715	−0.797	0.003	1.1
20	Dependency	.844	1.024	−0.865	0.000	0.4
21	Well-being, distress	.693	0.674	−0.836	0.000	0.4
22	Well-being, distress	.873	1.266	−0.331	0.004	0.7
23	Dependency	.766	0.878	−1.026	0.001	0.0
24	Dependency	.847	1.338	−0.904	0.000	0.0
25	Well-being, distress	.725	0.849	−0.822	0.015	0.4
*2	General vision	.891	0.531	−0.371	0.039	5.8
*3	Near vision	.897	1.111	0.084	0.138	2.2
*4	Near vision	.909	1.612	−0.285	0.509	12.3
*5	Near vision	.282	0.285	−2.196	0.014	1.1
*6	Distance vision	.779	0.863	−0.431	0.397	6.9
*7	Distance vision	.588	0.366	−0.593	0.016	38.4
*8	Distance vision	.758	1.137	−0.670	0.344	1.8
*9	Social functioning	.413	0.719	−1.884	0.000	5.8
*11A	Role limitation	.597	0.836	−1.226	0.057	0.4
*11B	Role limitation	.776	0.983	−0.951	0.001	1.1
*12	Well-being, distress	.791	0.653	−0.780	0.000	0.0
*13	Dependency	.422	0.432	−1.616	0.230	0.0
Deleted items						
1	General health	.208				0.4
4	Ocular pain	.059				0.4
10	Peripheral vision	.142				2.2
12	Color vision	.395				9.8
19	Ocular pain	.189				0.0
*1	General health	.265				2.9

*: optional items.

**: p value from χ^2^ test.

Factor analysis of the data from those 32 items resulted in the following eigenvalues for the first four factors: 18.16, 1.66, 1.39, and 1.18. More than half of the variance (59.4%) was explained by a one-factor solution. Factor loadings ranged from 0.907 to 0.342, and for 26 of the 32 items the loadings were 0.6 or greater. Because those findings strongly indicate that the scale can be considered to be unidimensional, we proceeded with the IRT-based analyses (Table 1).

### Selection of items

For the “general vision” domain, the item with the steeper slope was chosen (item 2; slope: 0.858). For the other domains, we first eliminated the 9 items with slopes less than 0.8, and then the 3 items with more than 20% missing data. Finally, considering each of the remaining 18 items' content, slope, location, and percentage of missing data, we chose at least one item for each domain. The 11 items thus chosen are indicated with asterisks in Table 2, and the instrument composed of them is the short-form VFQ 11-item Japanese version (VFQ-J11).

**Table 2:** Candidate items, and the 11 items chosen (*) for the short version of the VFQ (the VFQ-J11).

Domain	Number of items	Item numbers^a^
General vision	1	2*
Near vision	4	5*, 6*, optional-3*, optional-4
Distance vision	3	8*, optional-6*, optional-8*
Dependency	3	20, 23, 24*
Social functioning	2	11*, 13
Well-being, distress	2	22, 25*
Role limitation	3	17*, optional-11A, optional-11B

a See Table 1 for item numbers.

VFQ: The Visual Function Questionnaire originally developed by the National Eye Institute.

VFQ-J11: The short-form VFQ 11-item Japanese version.

### Targeting and Test Information

**Figure 1:**
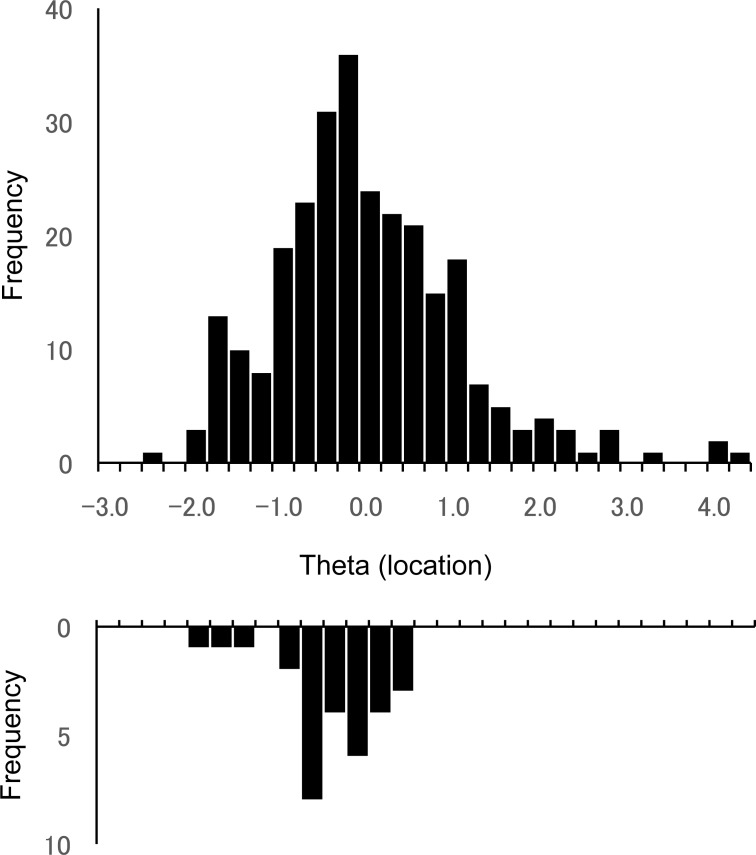
Targeting of item thresholds with regard to the patients in this study. The Figure shows locations of the patients (above) and of the item thresholds (below) on the same scale, in units of the latent trait.

Figure 1 shows the patients (above) and the thresholds from items (below) on the same scale, in units of the latent trait. The average of the items' locations was −0.73 (SD = 0.53), and the mean of the patients' thetas was −0.16 (SD = 1.07). As shown in Figure 1, the targeting of the item thresholds was not markedly skewed with regard to the patients in this study.

**Figure 2:**
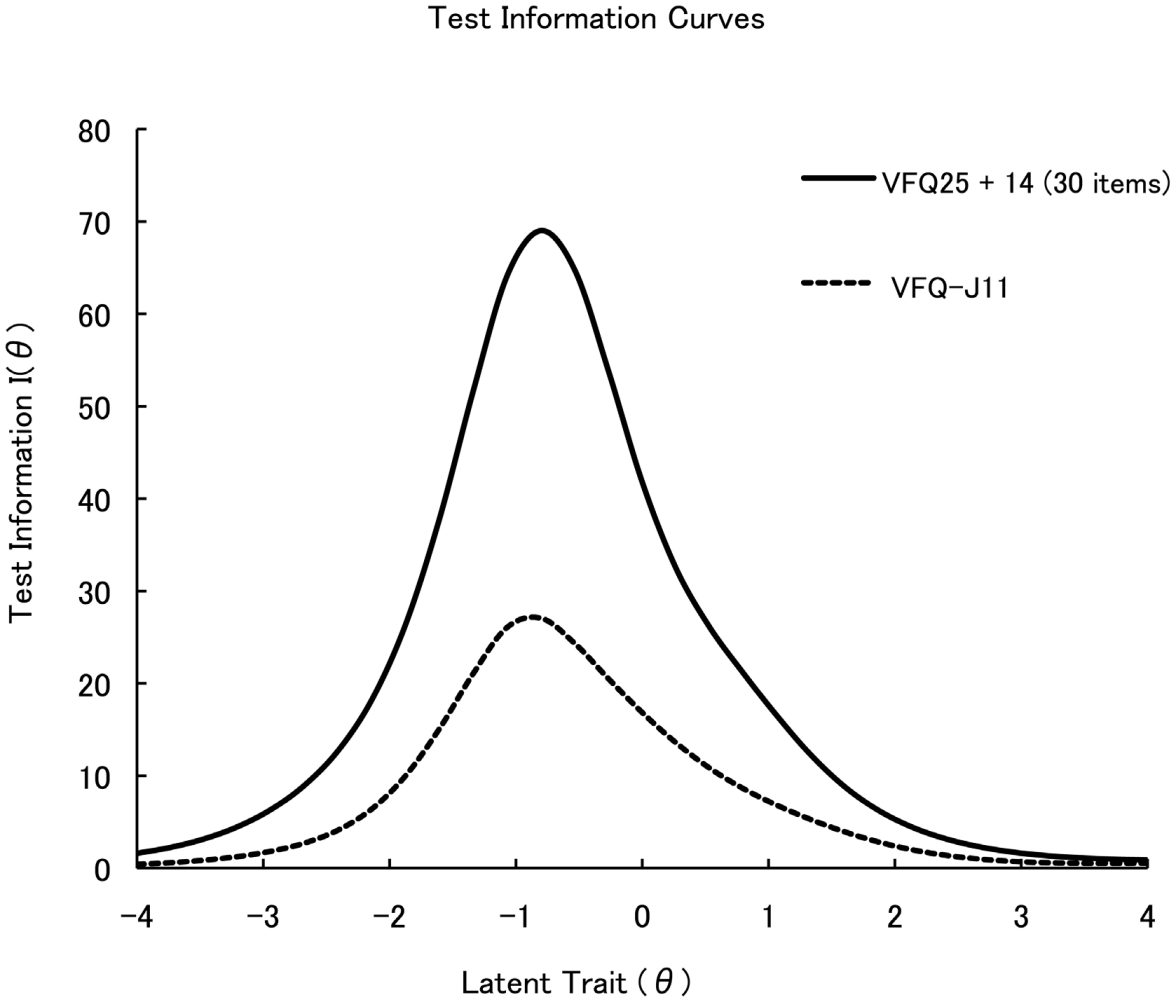
Test information functions for the VFQ25 and the VFQ-J11. Both functions reached their maxima at about the same value of the latent trait (a theta of approximately −0.8). For values of theta between approximately −2.0 and +0.8, the test information for the VFQ-J11 was greater than 9, which indicates that in that range (−2.0 to +0.8) the test's reliability is equivalent to or greater than a Cronbach's alpha of 0.9.

For values of theta between approximately −2.0 and +0.8, the test information for the VFQ-J11 (Figure 2) was greater than 9, which indicates that in that range (−2.0 to +0.8) the test's reliability is equivalent to or greater than a Cronbach's alpha of 0.9. For the VFQ-J11 as a whole, Cronbach's alpha was 0.94.

The test information functions for the VFQ25 and the VFQ-J11 (Figure 2) reached their maxima at about the same value of the latent trait (a theta of approximately −0.8). That is, the range in which the VFQ25 is most precise is maintained in the VFQ-J11. Using the mean and standard deviation of the VFQ-J11 scores measured in this study (63.41 and 24.54, respectively, Table 3), and the equation theta  =  (score – mean score)/standard deviation, we compute that the most precise measurements were at and near VFQ-J11 scores of approximately 44.

**Table 3:** Scores on the VFQ25 and VFQ-J11 in patients with different eye diseases.

Eye disease		VFQ25	VFQ-J11
	n	Mean ± SD	Mean ± SD
1. None	31	86.51±9.51	88.79±10.21
2. Glaucoma	69	72.33±17.35	73.63±19.07
3. Cataract	96	66.29±16.32	64.85±17.20
4. Age-related macular degeneration	80	48.75±21.70	43.02±24.92
All groups	276	64.99±21.32	63.41±24.54

Results of one-way ANOVA among the four diagnosis groups were as follows: F(2, 272)  = 41.61 for the VFQ25, and 52.35 for the VFQ-J11. Post-hoc tests by Tukey's method resulted in p values less than 0.05 for all comparisons *except* one: the comparison of VFQ25 results between group 2 and group 3.

VFQ25: The 25-item Visual Function Questionnaire originally developed by the National Eye Institute.

VFQ-J11: The short-form VFQ 11-item Japanese version.

### Criterion-based validation testing and effects of cataract surgery

Coefficients for the correlations of VFQ25 and VFQ-J11 scores with the corrected visual acuity and visual field measures are shown in Table 4. For both the better eye and the worse eye, the correlation between visual acuity and VFQ-J11 score was strong. The correlation between field of vision and VFQ-J11 score was also strong.

**Table 4:** Correlations of scores on the VFQ25 and the VFQ-J11 with visual acuity and with visual field.

	n		VFQ25	VFQ-J11
		Better eye	0.669	0.719
Visual acuity	271			
		Worse eye	0.611	0.659
		Better eye	0.498	0.513
Field of vision	63			
		Worse eye	0.391	0.417

VFQ25: The 25-item Visual Function Questionnaire originally developed by the National Eye Institute.

VFQ-J11: The short-form VFQ 11-item Japanese version.

The VFQ-J11 and VFQ25 scores for groups defined by diagnosis are shown in Table 4. One-way ANOVA was done with diagnosis as the independent variable (4 levels), after which Tukey's method was used for post-hoc comparisons. For VFQ25 scores, F(3, 272)  = 41.61, and the differences between pairs of diagnoses were significant for all pairs other than glaucoma-cataract. That is, there was no significant difference in VFQ25 scores between patients with glaucoma and patients with cataract. For VFQ-J11 scores, F(3, 272)  = 52.35, and the differences were significant for all pairs of diagnoses, including glaucoma-cataract.

Cohen's d for the differences between preoperative and postoperative VFQ-J11 scores was 0.99. Regarding EQ-5D scores the change was slightly smaller: 0.88. That is, after surgery the VFQ-J11 scores had improved by almost one standard deviation, and the VFQ-J11 was slightly more sensitive to change than was the EQ-5D.

## Discussion

We found that the VFQ-J11 was superior to the VFQ25 in terms of responsiveness and criterion-related validity. Because its length is less than half that of the VFQ25, the VFQ-J11 should also impose less of a burden on patients with vision problems.

We used IRT to develop this short version of the Visual Function Questionnaire. The new scale, the VFQ-J11, comprises 11 items from the original VFQ (which had a total of 39 items, when the 14 optional items are included). Without IRT, our decisions about which items to include in the VFQ-J11 would have been less well informed. Specifically, estimates of each item's location parameter indicated the level of visual function that each item measured best, and estimates of each item's slope parameter indicated the precision of that item's measurement. This new short version of the VFQ performed as well as, or better than, the original longer version.

With regard to precision, we note that both the long and the short versions were most precise near theta values of approximately −0.8, which corresponds to a VFQ-J11 score of approximately 44. Thus, the VFQ-J11 is particularly appropriate in evaluations of the functioning of patients whose disease affects them severely, such as those with age-related macular degeneration, who had a mean score of 43.

Regarding the data collected before and after cataract surgery, values of Cohen's d greater than 0.8 are generally taken to indicate the presence of “large” effects [22], and the instruments used here showed such large effects. However, the interpretation of these results is certainly limited because this small sample (n = 14) may not be representative of all patients in Japan who have cataract.

Correlations with results of tests of visual acuity and visual field were used for criterion-based validation testing, and data from the VFQ-J11 were more strongly correlated with those external criteria than were data from the VFQ25. In addition, scores on the VFQ25 did not distinguish patients with glaucoma and patients with cataract, whereas the VFQ-J11 scores of those two groups were significantly different. These results can be interpreted as meaning that the VFQ-J11 retains items that are relatively good indicators of vision-related QOL, and they indicate success of these validation tests. Also, as expected for a valid measure of visual function, patients with macular degeneration had very low scores.

In any psychological testing, we must handle problems caused by missing data. Data may be missing because, for example, the respondent does not understand the question or because the respondent finds the question to be inappropriate. The performance of a psychometric instrument will suffer if the respondents are not able to answer the questions and if they do not interpret the questions correctly. As much as possible for the VFQ-J11, we chose questions that could be answered by all potential respondents.

We note that this short-form instrument incorporates information about multiple domains into a single score. This may prove to be useful in future comparisons with other summary indicators, including utility measures. The practical advantages of a short form are also noteworthy. Especially for people with vision problems, answering 25 questions could be difficult, and we developed this short version to give them a task that is easier. Having fewer than half of the questions of its predecessor, the VFQ-J11 should be much less burdensome. In addition, we can expect those burdens to be further reduced by the relatively uniformity of the response format. Specifically, with “yes-no” questions, questions regarding frequency, and questions regarding severity, the VFQ25 has a total of eight different response formats, whereas the VFQ-J11 has only five.

In 2010, Pesudovs et al. reported on the development of short-form scales from the NEIVFQ. The original item pool used in those analyses included all 39 NEIVFQ items, but in the present analyses the original item pool did not include the three items related to driving. The data used in those analyses were obtained from patients, mainly elderly, in Australia who had cataract, but the data used to develop the VFQ-J11 were obtained from people in Japan, 35% of whom had cataract and 11% of whom had no chronic ophthalmologic disease, and who were, overall, somewhat younger. There may also be differences between Australian and Japanese people with regard to lifestyle-related effects of impaired vision on daily life. With those differences in mind, the comparisons below, between the scales developed by Pesudovs et al. and the VFQ-J11, should be interpreted only with caution.

One important difference between the results reported by Pesudovs et al. and the VFQ-J11 is that in the former there are separate “visual functioning” and “socioemotional” scales whereas the latter is unidimensional. The 36 items from which the VFQ-J11 was developed were clearly unidimensional, as indicated by the pattern of eigenvalues obtained by factor analysis. Items with low factor loadings from the domains of color vision, peripheral vision, and ocular pain were then identified and those items were not used in the short-form instrument. Similarly, the short-form scales developed by Pesudovs et al. also do not contain items intended to measure those domains. The VFQ-J11 uses 3 of the optional items from the original VFQ, while the short-form scales developed by Pesudovs et al. do not. Nonetheless the VFQ-J11 and the 13 items used by Pesudovs et al. do have 6 items in common. Regarding the domains that the items were originally intended to measure, the VFQ-J11 includes 3 distance-vision items while the 13 items used by Pesudovs et al. include 2 distance-vision items. The former includes 1 dependency item, 1 well-being & distress item, and 1 role-limitation item, while the latter includes 2 items from those 3 domains. However, both the VFQ-J11 and the 13 items used by Pesudovs et al. include 1 general-vision item, 3 near-vision items, and 1 social-functioning item.

The measurement model of the VFQ-J11 has a single dimension that is represented by 11 items, as noted above, which are “located” rather close to each other (Figure 1); and this precision of the VFQ-J11 has an implication regarding its utility. Specifically, the VFQ-J11 may be useful in situations that require small differences in visual functioning to be distinguished reliably. Examples of such situations could include interpreting an individual's data, and measuring changes over time, whether such changes occur in the natural course of a disease or after a therapeutic intervention.

One of the strengths of this study is the diversity of the participants. Three different important and chronic ophthalmologic conditions were represented, which should give clinicians some confidence that the VFQ-J11 will be useful among many of their patients. The use of IRT-based analyses is also a strength, because of the advantages, noted above, of IRT over CTT. Another strength is the demonstration that VFQ-J11 scores were responsive to the effects of cataract surgery.

The VFQ-J11 provides a relatively large amount of information about people who are severely affected by vision problems, and relatively little information about people whose visual functioning is closer to normal. Thus it is appropriate for ophthalmology patients in Japan, but it will give precise information about general-population samples only after it is supplemented with items targeted at the levels of visual functioning that are common in such groups. In addition, the VFQ-J11 does not include items asking about color vision, ocular pain, or peripheral vision, so clinicians and researchers interested in those areas will need to use other instruments to measure them.

Further work is required to establish appropriate criteria or an appropriate framework with which to interpret VFQ-J11 scores from patients' perspectives. That is, it is not yet sufficiently clear how those scores are related to patients' experiences using their vision in daily life. Qualitative studies might be helpful in that regard. Further validation testing should also include tests of responsiveness to interventions other than cataract surgery, and they should include tests of the VFQ-J11 as a stand-alone instrument, that is, without patients also being exposed to other VFQ items. As a more objective measure of the burdens imposed by VFQ instruments of different lengths, it would be useful in future studies to record the time required to complete each instrument.

### Summary

We found that the VFQ-J11 had high levels of test information in a range that is clinically relevant. We also found that it was more strongly correlated with visual acuity and with visual field than was the VFQ25. Unlike VFQ25 scores, VFQ-J11 scores were different between patients with glaucoma and patients with cataract. After cataract surgery, VFQ-J11 scores changed in the expected direction, and by a large amount.
